# Differential secretome of pancreatic cancer cells in serum-containing conditioned medium reveals CCT8 as a new biomarker of pancreatic cancer invasion and metastasis

**DOI:** 10.1186/s12935-019-0980-1

**Published:** 2019-10-11

**Authors:** Peng Liu, Lingming Kong, Haoyi Jin, Yunhao Wu, Xiaodong Tan, Bing Song

**Affiliations:** 1 1st Department of General Surgery, Shengjing Hospital, China Medical University, Shenyang, 110004 China; 2 Cardiff Institute of Tissue Engineering and Repair, School of Dentistry, Cardiff University, Cardiff, CF14 4XY UK

**Keywords:** CCT8, Pancreatic cancer, Serum-containing conditioned medium, Biomarker

## Abstract

**Background:**

Pancreatic cancer is a malignancy with a very poor prognosis. The emergence of liquid biopsy is expected to achieve accurate early diagnosis through detection of tumor-derived secreted proteins in the blood. Early diagnosis and treatment of pancreatic cancer could help to improve prognosis.

**Methods:**

The pretreatment approach of samples can have a major effect on downstream analysis. In this study, we used a pair of homologous pancreatic cancer cell supernatants with different capacities for invasion and metastasis to examine secreted proteins in the conditioned media without the removal of fetal bovine serum, namely through size exclusion chromatography combined with high-abundance protein affinity chromatography to enrich low-concentration protein, followed by mass spectrometry using triple dimethyl labeling. Identification of proteins was performed using an online public database and western blot.

**Results:**

Mass spectrometry data revealed 77 proteins with quantitative properties, of which 12 proteins had over a 1.5-fold difference (in the supernatant of the highly invasive pancreatic cancer cell line PC-1.0, the expression of 8 proteins were increased and the expression of 4 proteins were decreased). Bioinformatics analysis results showed that CCT8, CTSL, SAA1, IGF2 are secreted via the exosome pathway. According to the literature, with the exception of CCT8, the other three proteins can be detected in blood samples of pancreatic cancer patients, and they can be used as prognostic markers. Western blot results were used to validate consistency with MS results.

**Conclusion:**

This study found that CCT8 can be used as a liquid biopsy marker to assess the prognosis of pancreatic cancer patients.

## Background

Pancreatic cancer ranks fourth in cancer mortality in the United States. In 2019, 56,770 new cases of pancreatic cancer are expected in the United States. Due to its anatomical location deep in the abdominal cavity, patients do not present unique symptoms, and most tumors are at an advanced stage at the time of diagnosis and are often accompanied by vascular invasion or distant metastasis. Surgical treatment is an option in only 15–20% of pancreatic cancer patients, and the overall 5-year survival rate is less than 5% [1, 2]. Early diagnosis and appropriate treatment can significantly improve the prognosis of pancreatic cancer. With the advancement of experimental techniques, the number of molecular and chemical detection methods for pancreatic cancer has increased, and these methods have played an important role in the early diagnosis of pancreatic cancer [3].

Tumor microenvironment can regulate cancer development and metastasis. Hanahan et al. have reported that heterogeneous signaling pathways between the parenchyma and mesenchyme of a tumor play an important role in tumor invasion and metastasis [4]. Studies have confirmed that secreted proteins play an important role in the tumor microenvironment [5]. In previous studies, we used two cell lines with distinct capacities for invasion and metastasis: a non-dissociated, weakly metastatic pancreatic cancer cell line (PC-1) and a dissociated, highly metastatic pancreatic cancer cell line (PC-1.0). PC-1.0 cell supernatant was purified, prepared into a conditioned medium, and used for culture of the weakly metastatic PC-1 cells. It was found that the growth of PC-1 cells changed to the growth pattern of PC-1.0. Therefore, we concluded that PC-1.0 supernatant contains key factors that promote changes in the biological behavior of the cells [6].

In this study, the secretome was extracted from the serum-containing supernatant, and low-abundance proteins were enriched by online chromatographic separation. In addition, the pancreatic cancer secretome was analyzed in combination with triple dimethyl labeling. 77 proteins were identified, of which 12 with a fold change of over 1.5 were considered differentially expressed proteins. DAVID and STRING software were used to analyze the function of differential proteins and their possible interacting proteins. Survival analysis of these differential genes was performed using a public pancreatic cancer database. Finally, we selected CCT8 and CTSL for protein validation in two pairs of cell lines with different capacities for invasion and metastasis.

## Materials and methods

### Instruments, reagents, and materials

Acetonitrile (ACN) and methanol were purchased from Merck (Germany). Glacial acetic acid was purchased from Damao Chemical Reagent Factory (Tianjin, China). Bovine serum albumin (BSA), formaldehyde and its isotopic reagents (CH_2_O, ^13^CH_2_O, ^13^CD_2_O, CD_2_O), sodium cyanoborohydride (NaBH_3_CN), deuterated sodium cyanoborohydride (NaBD_3_CN), heavy water (D_2_O), α-cyano-4-hydroxycinnamic acid (CHCA), and trifluoro acetic acid (TFA) were purchased from Sigma-Aldrich (USA). All water for experiments was purified using the Milli-Q system (Millipore, USA). The centrifuge was purchased from Sigma (USA). The Thermo SEC120 HPLC column (size 5 μm, 120 Å) and Agilent Human 14 Multiple Affinity Removal Column (H14, size: 10 mm × 100 mm) were used. The Ultimate 3000 chromatography instrument was used with the Thermo LTQ-Orbitrap mass spectrometer for detection.

### Cell culture

PC-1.0 and PC-1 cells were given friendly by Kumamoto University Medical School (Kumamoto, Japan) and the use of these cell lines was approved by China Medical University Affiliated Shengjing Hospital Medical Research. Aspc-1, Capan-2 cells, and HPDE6-C7 were purchased from the Institute of Biochemistry and Cell Biology, Chinese Academy of Sciences (Shanghai, P.R. China). PC-1 cells grew as islet-like clones of cells, whereas PC-1.0 cells grew as single cells. These cells were cultured in RPMI-1640 (Gibco-BRL, Grand Island, NY) supplemented with 10% fetal bovine serum + 1% streptomycin. Cells were incubated in a 37 °C incubator with 5% CO_2_. Cells were passaged when they reached a confluence of about 80% [7].

### Experimental methods

#### Extraction of total protein from samples

**Fig. 1:**
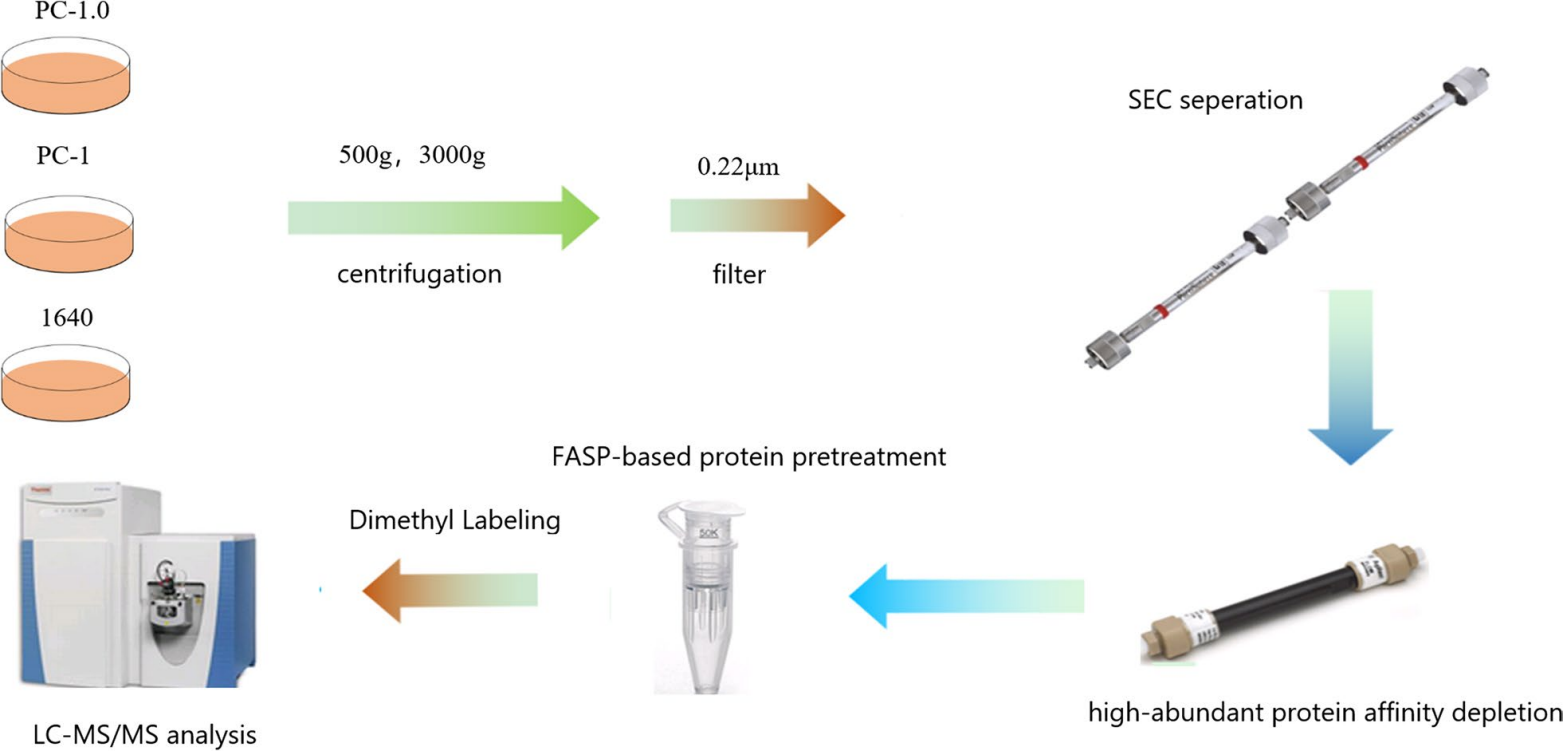
Conditioned media of PC-1.0 derived, PC-1 derived, and 1640 were validated by SDS-PAGE

PC-1.0 and PC-1 cells were cultured in complete growth medium until 80–90% confluent. The supernatant was collected and protease inhibitors were added. The supernatant was centrifuged at 500*g* and 3000*g* for 15 min to remove debris. The mixture was spun at 12000 r/min through a 0.22 μm fiber filter and concentrated using a 3 kDa concentrating tube by centrifuging at 3500*g* for 120 min. Protein samples included three independent biological replicates. Protein concentration was measured using the BCA method. The experimental process is shown in Fig. 1.

#### SEC and H14 sample isolation

High-abundance proteins were removed using a size exclusion chromatography (SEC) column combined with an Agilent multiple affinity removal spin cartridge (H14) to enrich low-abundance proteins. A 200 μL sample was injected and washed with buffer A at a flow rate of 0.5 mL/min for 10 min. After the flow through fractions were collected, the remaining fractions were eluted with buffer B at 1 mL/min for 7 min. The collected fractions were stored at − 20 °C until use. The flow through fractions collected from the injection were concentrated in a rotary concentrator with a 5 kDa molecular weight cut-off membrane. The sample was centrifuged at 5000 r/min at 10 °C.

#### Triple dimethyl labeling

Protein concentration was measured using the BCA method, and samples were prepared using FASP. PC-1.0 cells were labeled with 50 mM pH 8.0 phosphate buffer with 20 μL of 4% formaldehyde and 20 μL of 0.6 M sodium cyanoborohydride (light label) added. PC-1 cells were labeled with 20 µL 4% deuterated formaldehyde and 20 μL of 0.6 M sodium cyanoborohydride (medium label). 1640 medium was labeled with 20 μL of 4% ^13^CD_2_O and 20 μL of 0.6 M deuterated sodium cyanoborohydride (heavy label). The reaction was conducted at room temperature for 1 h. Labeled solutions were combined, lyophilized and stored at -20 °C.

#### Capillary liquid chromatography-mass spectrometry analysis and database search

The Ultimate 3000 chromatography instrument was used with the Thermo LTQ-Orbitrap mass spectrometer. The flow direction was divided into an A phase (98% H_2_O and 2% ACN with 0.1% formic acid) and a B phase (2% H_2_O and 98% ACN with 0.1% formic acid). We used a custom C18 capillary trap column (150 μm inner diameter × 4 cm) and a separation column (75 μm inner diameter × 15 cm) with a flow rate of 120 μL/min. Mobile phase setting: 0–6% B phase for 10 min, then 6–35% B phase for 100 min, 35–80% B phase for 10 min, and finally 80% B phase for 10 min. The voltage was set to 2.7 kV and the ion transfer capillary temperature was 275 °C. Mass spectrometry was sequenced in a data-dependent manner. The CID ion dissociation method was used, a dynamic 20-second exclusion window was set, and the mass spectrometry ion mass-to-charge ratio scan range was from 300 to 1800. Samples were measured in triplicate. The experimentally obtained mass spectra were searched using Maxquant software to analyze the data files (.RAW) produced by the mass spectrometry. Settings for relevant parameters: mass error of the parent ion was 7 ppm, and mass error of the daughter ion was 20mmu. The fixation modification was an alkylation modification to cysteine, and the variable modification is an oxidation modification of methionine. The maximum number of missed sites allowed was 2, and the FDR for peptide and protein identification was less than 1%.

#### Bioinformatics analysis

In this study, quantitative data was obtained for all three experiments, the *p*-value was < 0.05, indicating that the data was reliable. We considered fold differences greater than 1.5 to be differential expression. To elucidate the function of secreted proteins, we performed bioinformatics analysis using DAVID (http://david.abcc.ncifcrf.gov/) [8] and STRING (https://string-db.org/) [9] software. In addition, we also searched the Exocarta database [10] to determine if these proteins were exosomes. Finally, we performed a matching search on pancreatic cancer genomics studies publicly available so far, and observed the association of genes corresponding to these proteins with pancreatic cancer [11].

#### Sodium dodecyl sulfate polyacrylamide gel electrophoresis (SDS-PAGE)

To an appropriate amount of protein sample solution, an equal volume of reducing loading buffer (2% SDS, 20% glycerol, 20 mmol/L Tris–HCl, pH 6.8, 6 mmol/L ß-mercaptoethanol, a trace amount of bromophenol blue) was added. After boiling for 5 min, separation was performed using a vertical electrophoresis apparatus. After the electrophoresis was finished, the film was stained with silver nitrate and scanned.

#### Western blot

Western blotting was performed as described previously [7]. Primary antibodies against CCT8 polyclonal antibody (Abcam, USA) and CTSL polyclonal antibody (Proteintech, USA) were used. Supernatant was collected when the cells reached 80–90% confluence, and protease inhibitors were added. The supernatant was centrifuged at 500*g* and 3000*g* for 15 min, the supernatant was collected, proteins were concentrated using a 3 kDa concentrating tube, and protein concentration was measured using the BCA method. Samples of equivalent total protein (20 μg) were loaded.

#### Cell transfection

shRNA plasmid specific for CCT8, scrambled shRNA, and lentiviral vector were purchased from GeneChem (GeneChem, Shanghai, China). Stable transfection with lentiviral vector was performed in accordance with manufacturer’s protocols. Scrambled shRNA was used as control.

#### Cell invasion and migration assays

Wound healing migration assay and Transwell invasion assay were performed as previously described [7].

## Results

### Immunoblotting detection of pancreatic cancer supernatant

**Fig. 2:**
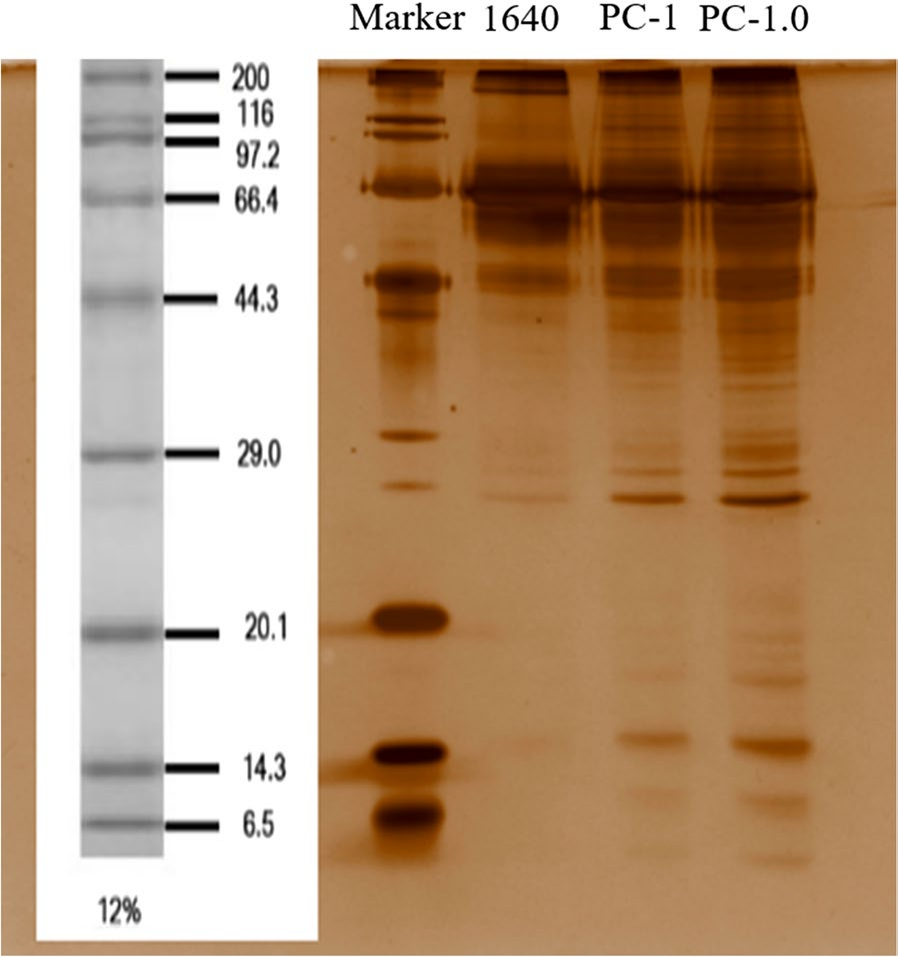
PPI network analysis of differentially expressed secretome proteins from PC-1.0 and PC-1 cells

In order to initially detect overall protein content in the supernatant of pancreatic cancer cells, we collected total protein from the supernatant and performed SDS-PAGE electrophoresis and silver staining (Fig. 2). The results showed that the approximate distribution of proteins in the supernatant of pancreatic cancer cells: high molecular weight proteins (greater than 44 kDa) were abundant, and there were fewer low molecular weight proteins (less than 20 kDa).

### Proteins with quantitative differential expression

The experimental samples were separated using SEC and H14 columns to enrich the concentration of low abundance proteins. A total of 77 proteins were identified using Maxquant software through the combined calculations of three experiments (Additional file 1: Table S1 and Additional file 2: Table S2). Functional annotation analysis of these proteins using DAVID revealed close association with cell secretion, cell proliferation, and migration and metastasis (Table 1). In the experiment, we performed triple dimethyl labeling, namely, supernatants of highly invasive PC-1.0 cells with a light label, supernatants of weakly invasive PC-1 cells with a medium label, and 1640 medium with a heavy standard. By labeling the 1640 medium, we removed interference from serum proteins in the medium, which enhanced the reliability of the identification results. Based on the results of the search, we considered proteins with an over 1.5-fold change in the ratio of the three labels as significant differentially expressed proteins. The results showed that there were 12 differentially expressed proteins, of which 8 were up-regulated and 4 were down-regulated (Table 2).

**Table 1:** DAVID revealed close association with cell secretion, cell proliferation, and migration and metastasis

Category	Term	P-value	Fold enrichment
Annotation Cluster 1	Enrichment Score: 15.7		
UP_KEYWORDS	Secreted	5.81E−26	12.93408
Annotation Cluster 2	Enrichment Score: 3.3		
GOTERM_MF_DIRECT	GO:0031994 ~ insulin-like growth factor I binding	1.29E−04	163.6875
Annotation Cluster 3	Enrichment Score: 3.0		
KEGG_PATHWAY	bta04610:Complement and coagulation cascades	6.54E−05	21.25563
Annotation Cluster 4	Enrichment Score: 2.6		
UP_KEYWORDS	Extracellular matrix	1.02E−05	20.19811
Annotation Cluster 5	Enrichment Score: 2.5		
GOTERM_CC_DIRECT	GO:0005604 ~ basement membrane	0.009647	19.7711
Annotation Cluster 6	Enrichment Score: 1.9		
KEGG_PATHWAY	bta04510:Focal adhesion	0.003284	7.562099
Annotation Cluster 7	Enrichment Score: 1.8		
UP_SEQ_FEATURE	Domain:Peptidase S1	0.002328	14.57442

**Table 2:** List of 12 proteins differentially expression by PC-1.0 and PC-1 pancreatic cancer cells with fold change > 1.5

Protein	Ratio L/M (PC-1.0/PC-1)	Ratio L/H (PC-1.0/1640)	Ratio M/H (PC-1/1640)
SAA1	6.96	1.56	0.22
Roquin-1	3.29	1.52	0.55
CCT8	2.74	1.91	0.63
ZNF518B	2.14	4.39	1.85
CTSL	1.77	17.12	10.46
EXOSC8	1.60	3.16	2.57
IGF2	1.61	0.64	0.43
C4orf470	1.62	0.47	0.32
Npc2	0.57	8.55	13.36
HSP90AA1	0.45	0.66	1.54
PPIA	0.10	1.80	18.16
ENO1	0.05	1.89	31.58

### Gene ontology, KEGG, and PPI analysis

**Fig. 3:**
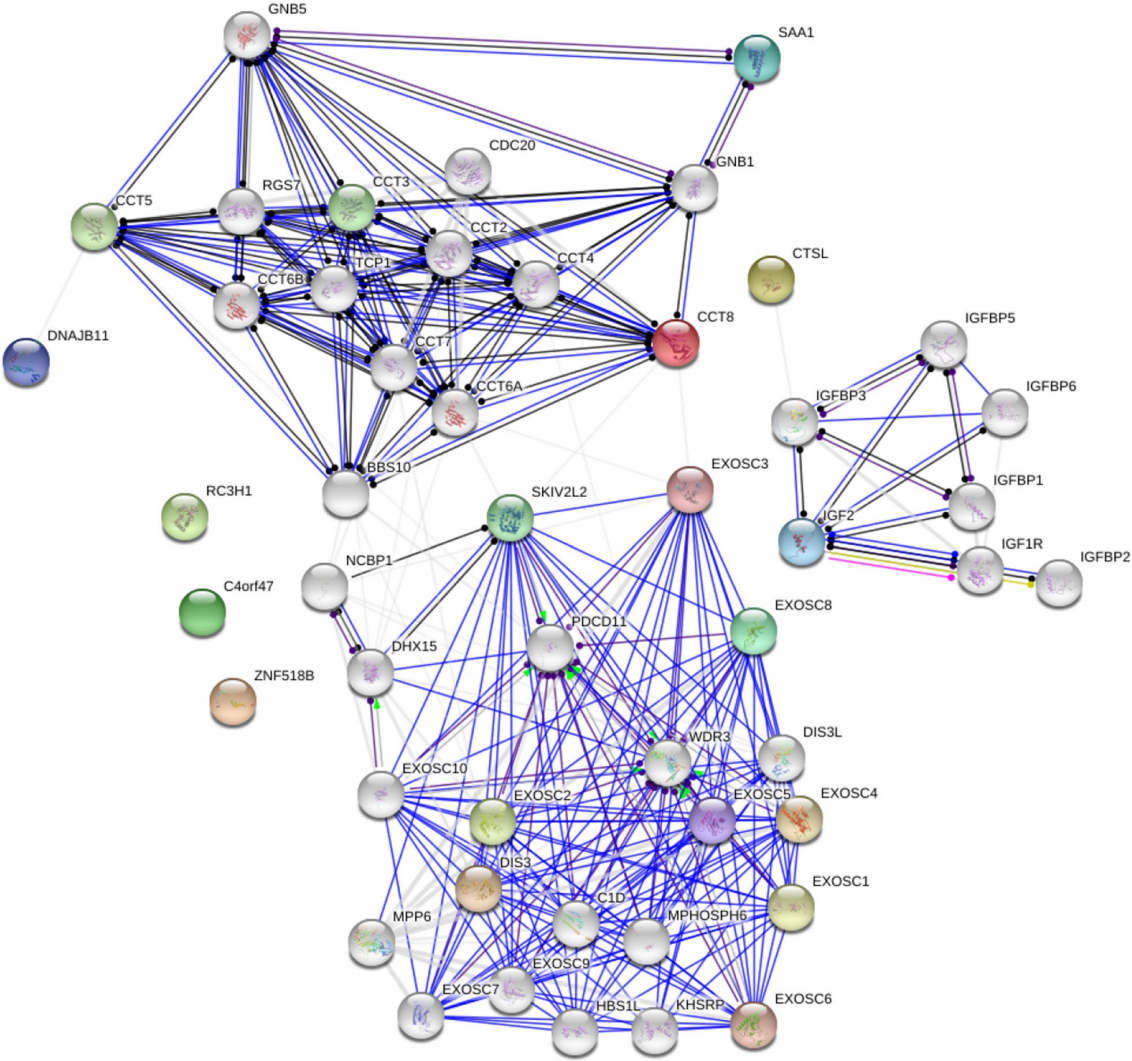
CCT8 is a key protein in this network

GO analysis was performed using online DAVID software. The primary enrichment in cellular component (CC) was cytoplasmic vesicle and extracellular exosome, and the primary enrichment in biological process (BP) was the regulation of cell adhesion. The primary enrichment in molecular function (MF) was protein complex binding. Using functional annotation clustering analysis in the software, we found that the primary cluster was exosome signaling, which included four proteins (CTSL, CCT8, IGF2 and SAA1). These results also confirmed that we identified secreted proteins (Additional file 3: Table S3). KEGG analysis showed that proteins were concentrated on the proteoglycans in cancer (*p* = 0.0025) signaling pathway. To further analyze the role of the identified proteins, we used the STRING database to search for DEPs. The results showed that there is currently no literature support for interaction between the eight proteins, which may be due to the small number of proteins we identified. Therefore, we used the increased protein interaction function built into the software. It was found that as the software gradually increased protein interaction, SAA1 and CCT8 could interact through GNB1, CCT8 and EXOSC8 were linked by EXOSC3, and CTSL and IGF2 were linked by IGFGB3. Therefore, through data mining, we believe that CCT8 may be a key protein in this PPI network (Fig. 3, Additional file 4: Table S4).

### Possible clinical significance of differential proteins

**Fig. 4:**
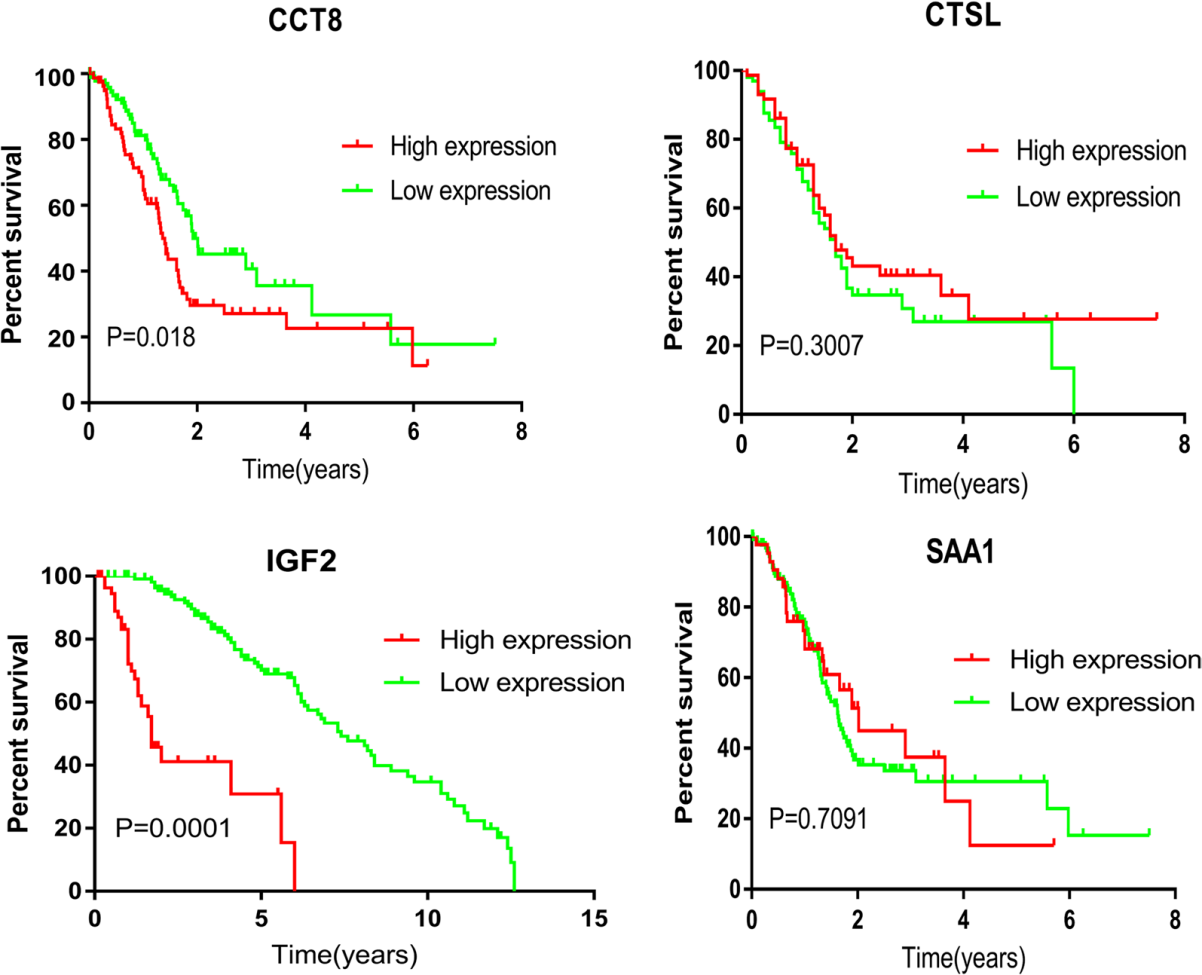
Expression level and prognostic value of the differentially expressed secretome proteins in Human Protein Atlas database

We used the Human Protein Atlas database to search CCT8, CTSL, IGF2, and SAA1 for clinical correlation analysis. Using clinical data on pancreatic cancer provided by Pathology Atlas, we analyzed the FRKM values of the four genes and found that CCT8 and IGF2 were associated with clinical prognosis (*p *= 0.018, *p *= 0.0001) (Fig. 4, Additional file 5: Table S5). There have been no literature reports of the function of CCT8 in pancreatic cancer. We searched the Exocarta database and our results from exosome studies, as well as these four proteins [12], which revealed from a different perspective that the four secreted proteins were secreted outside the cell in the form of exosomes. According to currently available public data, these four secreted proteins are present in human plasma samples and can be used as liquid biopsy markers in the future.

### Western blot validation of the expression of differentially expressed proteins in cell lines with different capacities for invasion and metastasis

**Fig. 5:**
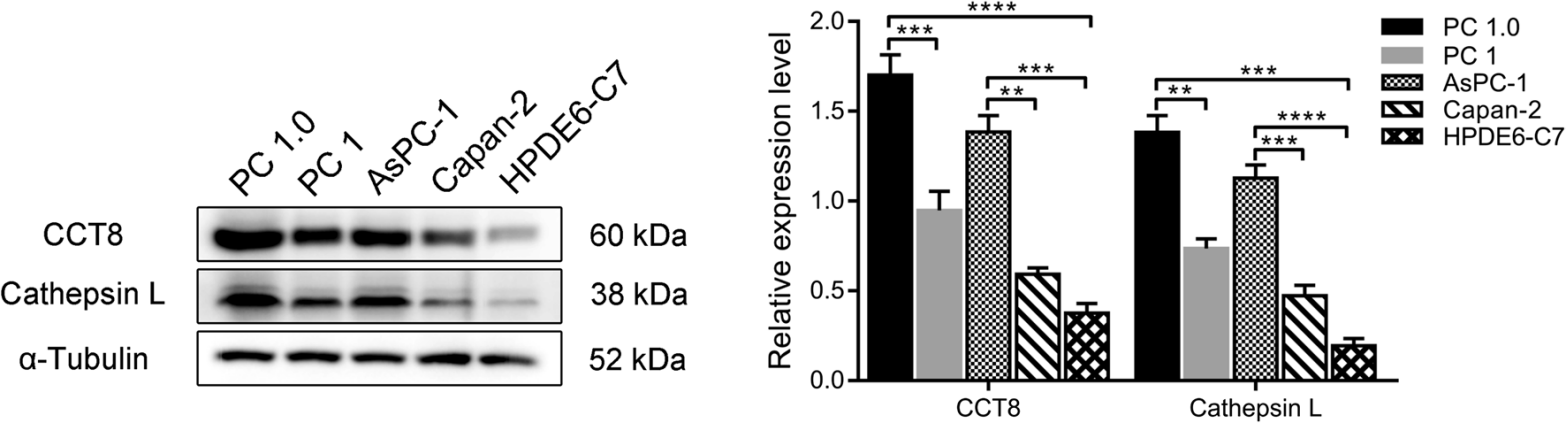
Western blot validation of CCT8 and CTSL from PC-1.0, PC-1, AsPC-1, Capan-2 and HPDE6-C7 cell lines (n = 3)

We used two pairs of cell lines (PC-1.0 vs. PC-1 and Aspc-1 vs. Capan-2) to measure intracellular CTSL and CCT8 protein expression. The results showed that CTSL and CCT8 were highly expressed in the highly invasive PC-1.0 and Aspc-1 cell lines (Fig. 5). This result is consistent with mass spectroscopy.

### Knockdown of CCT8 suppressed the migration and invasion capabilities

**Fig. 6:**
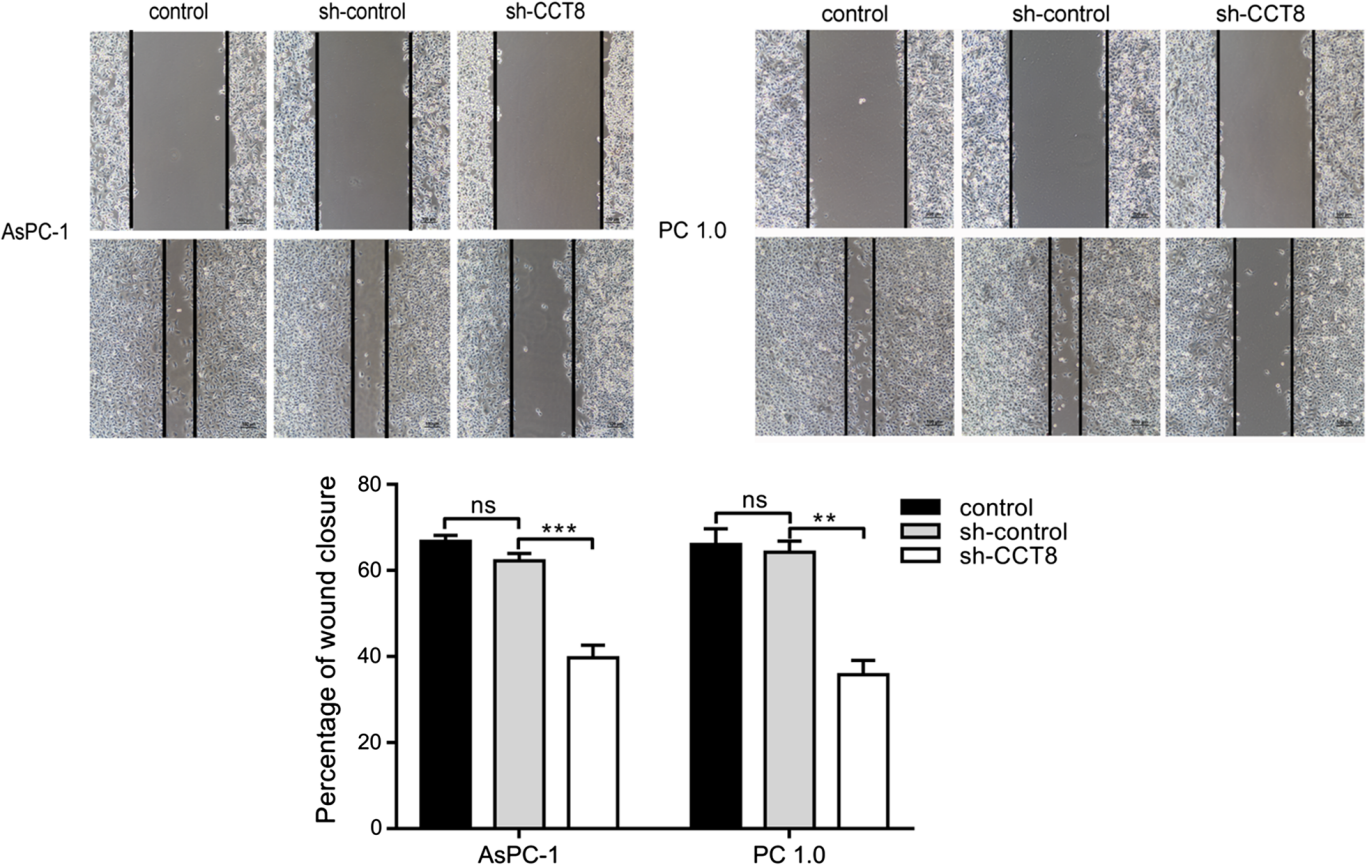
Knockdown of CCT8 suppress migration capability in PC-1.0 and AsPC-1 cell lines (magnification, ×100) (n = 3)

**Fig. 7:**
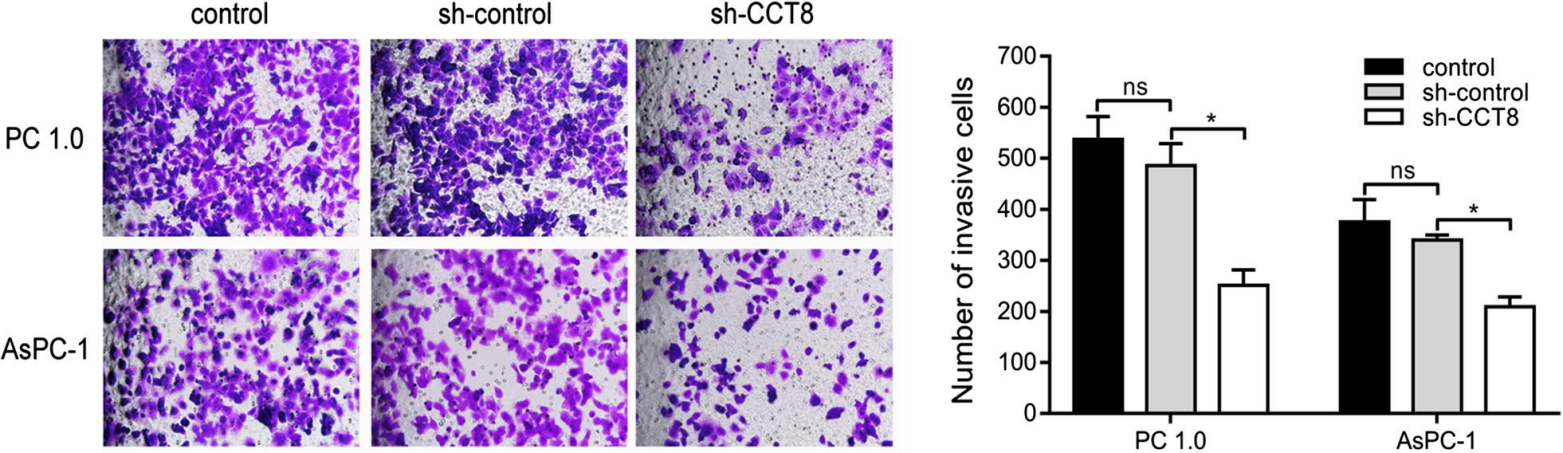
Knockdown of CCT8 suppress invasion capability in PC-1.0 and AsPC-1 cell lines (magnification, ×100) (n = 3)

To investigate the potential role of CCT8 in the metastasis of pancreatic cancer, we knocked down the expression of CCT8 in PC-1.0 and AsPC-1 cell lines. In vitro migration and invasion assay showed that knockdown of CCT8 suppressed the migration and invasion capabilities of PC-1.0 and AsPC-1 cells (Figs. 6, 7). These results suggested that knockdown of CCT8 could suppress the metastatic phenotype of these two pancreatic cancer cell lines.

## Discussion

**Fig. 8:**
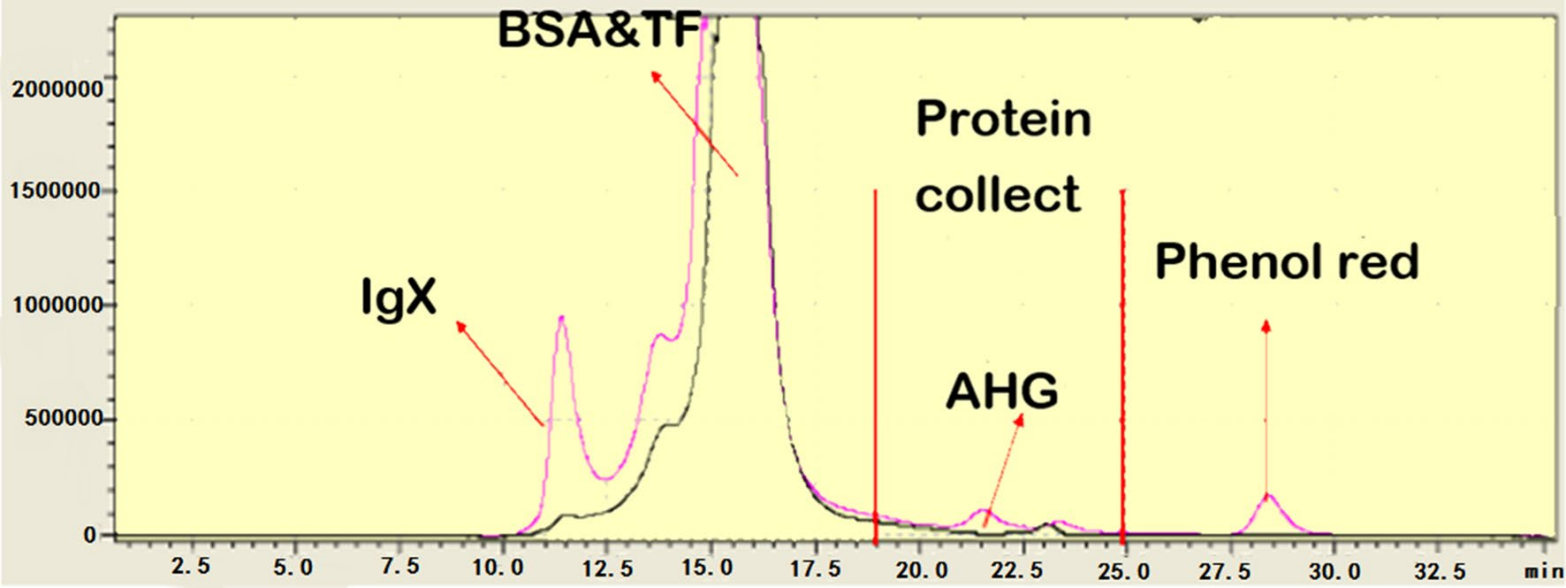
The figure show that the collection time of sample fraction in chromatography

When tumor cells secrete proteins into the extracellular environment, some of these proteins can alter the tumor microenvironment to promote tumor growth [13]. Previous studies have also confirmed some tumor biomarkers through secretome analysis. Most proteomic studies were performed in serum-free medium. These studies have used serum starvation to reduce interference from a large number of serum proteins and have found many biomarkers, but many significant limitations still exist [14, 15]. For example, many secreted proteins in culture media can cause changes in secretion status even during the short-term serum starvation process [16]. The pretreatment approach can have a major impact on downstream analysis. Therefore, we did not perform serum starvation in our experiments. In order to reduce the effects of high-abundance proteins, the secreted proteins in the supernatant were separated using the SEC and H14 columns, and the results were thus more reliable (Fig. 8).Based on the results of previous experiments, cell biological changes can occur between pancreatic cancer cells with different capacities for invasion. The difference between the secretomes produced by two cell lines is very important for understanding the molecular mechanism of tumor cell invasion and metastasis.

Through GO and KEGG analysis, it was confirmed that the main functions of the differential proteins were concentrated in cell secretion, which increased the reliability of our experimental results. It is well known that tumor development and progression occurs over three stages, of which cellular cancerous transformation is the best stage for monitoring and treatment. Liquid biopsy can be used to observe the risk of carcinogenesis at the cellular level, provide early warning, and be used for screening of high-risk populations. Traditional pathological biopsy has certain risks, and these tumor biomarkers derived from secreted proteins can be directly detected in body fluids, which can help distinguish between benign and malignant tumors earlier and more quickly, and help develop more appropriate treatment plans. In addition, they can also suggest the existence of occult micro-metastases in the body. For patients with metastatic tumors without metastasis or undiscovered metastasis, it can help with more accurate staging and guide clinical selection of more appropriate treatment methods [17].

To summarize the results of this manuscript, we have identified a small number of proteins, but the literature and our own data confirm that these differential proteins are secreted via exosomes, which proves that the results of experiments are close to the actual status of secretion. PPI network analysis showed that there was no direct relationship between these eight differential proteins. After data mining, we found that CCT8 is a potential core protein. Chaperonin-containing T-complex protein (CCT) has eight subunits, CCT1–8. The literature reports that CCT8 can affect invasion and metastasis of glioma cell carcinoma [18]. The TRic complex formed by CCT8 and other proteins can mediate protein folding and play an essential role in the telomerase pathway [19]. Currently, the relationship between the function of CCT8 as a secreted protein and pancreatic cancer has not been reported in the literature. We found through survival analysis that CCT8 can be used as a predictor of pancreatic cancer. CTSL upregulation has been found in many cancers to be positively associated with cancer invasion and metastasis and poor prognosis [20–23]. In addition, it has been reported in the literature that CTSL is highly expressed in the plasma of patients with pancreatic cancer and can be used as an independent predictor of pancreatic cancer [24]. Toriola et al. detected the expression of circulating IGF2 in blood, which was confirmed to be associated with pancreatic cancer prognosis [25]. SAA1 is highly expressed in cancer-associated fibroblasts (CAFs), and the higher the expression of SAA1 in the matrix composition, the worse the prognosis. Selective inhibition of SAA1 expression in CAF may provide potential therapeutic benefit to patients with PDAC [26]. In addition, detection of SAA in the blood is useful for distinguishing between malignant and benign diseases as well as healthy controls [27]. Online database suggested that CTSL and SAA1 were not associated with pancreatic cancer prognosis (*p* > 0.05), probably due to changes in protein content during tissue sample processing. Further in vitro functional experiments showed that knockdown of CCT8 suppressed invasion and metastasis of pancreatic cancer cell lines. In future studies, we will perform functional analysis of CCT8in vivo, while collecting clinical blood samples for cohort studies.

## Conclusion

In this study, samples were separated using two columns together in conjunction with dimethyl labeling, and mass spectrometry were used to improve the identification of secreted proteins under complete-medium conditions. Through biological analysis and cytological validation, it was confirmed that circulating CCT8 is a new predictive biomarker for pancreatic cancer.

## Supplementary information

**Additional file 1: Table S1.** A complete list of all the proteins identified in this study.

**Additional file 2: Table S2.** The acquired raw files were analyzed by Maxquant. List of 12 proteins differentially expression by PC-1.0 and PC-1 pancreatic cancer cells with fold change > 1.5.

**Additional file 3: Table S3.** GO term analysis of the identified proteins by DAVID.

**Additional file 4: Table S4.** String network coordinates.

**Additional file 5: Table S5.** The mRNA expression levels of 4 gene in pancreatic cancer were searched against the Human Protein Atlas data.

## Data Availability

All data generated or analyzed during this study are included in this published article and its additional files.

## Abbreviations

CCT8: chaperonin-containing T-complex protein 8
CTSL: cathepsin L
IGF2: insulin like growth factor 2
GO: gene ontology
SAA1: serum amyloid A1
ACN: acetonitrile
BSA: bovine serum albumin
TFA: trifluoroacetic acid
SEC: size exclusion chromatography
BCA: bicinchoninic acid
FASP: filter-aidedsample preparation,
CC: cellular component
BP: biological process
MF: molecular function
shRNA: short hairpin RNA
SDS-PAGE: sodium dodecyl sulfate polyacrylamide gel electrophoresis
